# Supplementary material for: Combinatorial Virtual Library Screening Study of Transforming Growth Factor-β2–Chondroitin Sulfate System
Source: Int J Mol Sci. 2021 Jul 14;22(14):7542. doi: 10.3390/ijms22147542 (PMC8305211; doi:10.3390/ijms22147542)
Supplement: Supplementary file 1 [file ijms-22-07542-s001.zip › TGFb_SI_7Jun2021.pdf]

SUPPLEMENTARY INFORMATION

Combinatorial Virtual Library Screening Study of Transforming Growth Factor Beta-2 – Chondroitin Sulfate System

Nehru Viji Sankaranarayanan[1,2#], Balaji Nagarajan,[1,2#] and Umesh R. Desai[1,2*]

[1]*Institute for Structural Biology, Drug Discovery and Development and* [2]*Department of Medicinal Chemistry, Virginia Commonwealth University, Richmond, VA 23298, USA*

[#]*Equal contributing authors*

*Addresses for correspondence: Umesh R Desai (urdesai@vcu.edu), 800 E. Leigh Street, Suite 212, Richmond, VA 23219. Ph (804) 828-7575; Fax (804) 827-3664

Table of Contents

#	Item	Pg. #
1	**Figure S1.** Sequence of TGF-β2 – to identify GAG binding sequence motif	S2
2	**Figure S2.** Conformational space exploration of TGF-β2-CS06 complex in binding site1 (BS1)	S3
3	**Figure S3.** Conformational space exploration of TGF-β2-CS06 complex in BS1	S4
4	**Figure S4.** Conformational space exploration of TGF-β2-CS06 complex in binding site 2 (BS2)	S5
5	**Figure S5**. Conformational space exploration of TGF-β2-CS06 complex in BS2	S6
6	**Figure S6**. Conformational sampling of two CS06 sequences simultaneously bound in BS1 and BS2 of TGF-β2	S7
7	**Figure S7**. Conformational sampling of two CS06 sequences simultaneously bound in BS1 and BS2 of TGF-β2	S8

```
10         20         30         40         50
MHYCVLSAFL ILHLVTVALS LSTCSTLDMD QFMRKRIEAI RGQILSKLKL
        60         70         80         90        100
TSPPEDYPEP EEVPPEVISI YNSTRDLLQE KASRRAAACE RERSDEEYYA
       110        120        130        140        150
KEVYKIDMPP FFPSENAIPP TFYRPYFRIV RFDVSAMEKN ASNLVKAEFR
       160        170        180        190        200
VFRLQNPKAR VPEQRIELYQ ILKSKDLTSP TQRYIDSKVV KTRAEGEWLS
       210        220        230        240        250
FDVTDAVHEW LHHKDRNLGF KISLHCPCCT FVPSNNYIIP NKSEELEARF
       260        270        280        290        300
AGIDGTSTYT SGDQKTIKST RKKNSGKTPH LLLMLLPSYR LESQQTNRRK
       310        320        330        340        350
KRALDAAYCF RNVQDNCCLR PLYIDFKRDL GWKWIHEPKG YNANFCAGAC
       360        370        380        390        400
PYLWSSDTQH SRVLSLYNTI NPEASASPCC VSQDLEPLTI LYYIGKTPKI   <- TGF-β2
       410
EQLSNMIVKS CKCS
```

Figure S1. The sequence of TGF-β2 extracted from Uniprot ID: P61812. The region of TGF-β2 is shown in gray. The mapping of potential GAG binding was done by considering the presence of Cardin and Weintraub consensus sequence motif ([–XBBXBX–] and [–XBBBXXBX–]; B = basic residue and X = hydropathic residue.). The sequence region belonging to TGF-β2 did not show any consensus sequence pattern. See Methods for details.

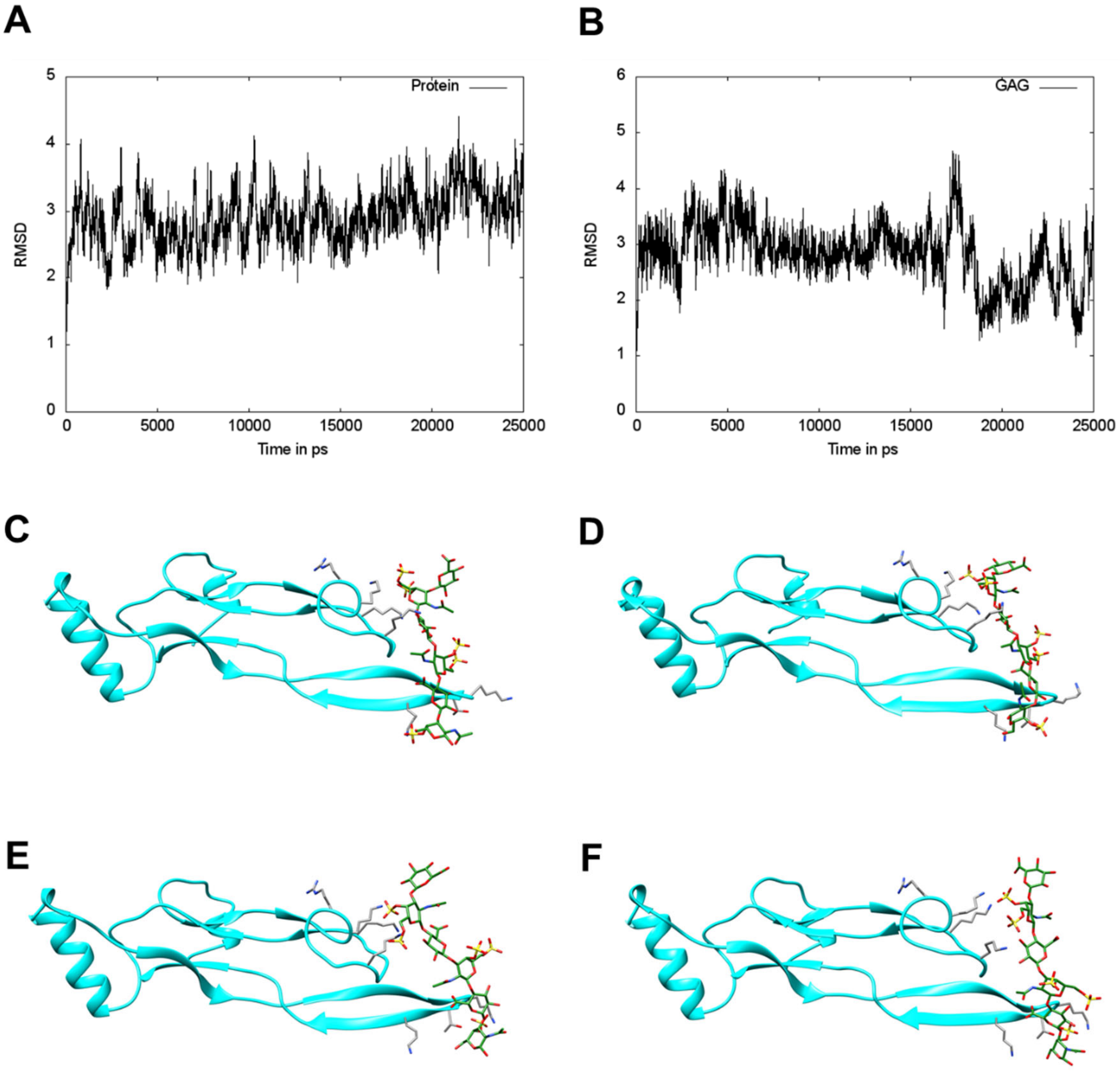

Figure S2. Conformational sampling of CS06 bound in BS1 of TGF-β2 from MD simulation to give an overall picture on the movement of protein (A) and ligand (B) was in presence of solvent (see **Movie SM1**). (C) through (F) show representative frames of the MD simulation showing the bound hexasaccharide (in green sticks) and the interacting residues (shown as sticks). See Methods for details.

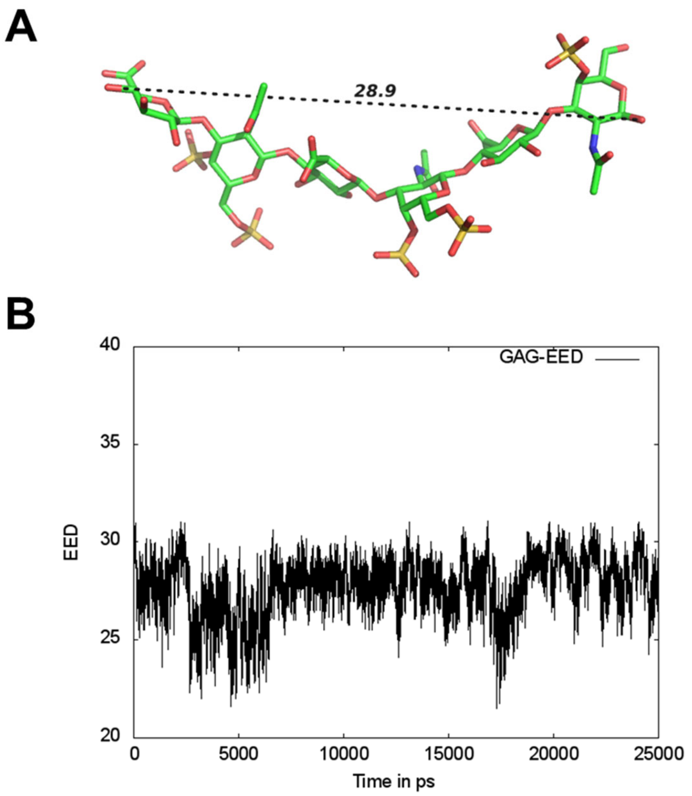

Figure S3. Conformational space exploration of TGF-β2-CS06 complex in BS1. A) A representative structure showing the end-to-end distance (EED) of the CS06. B) Time-based fluctuation of the EED during the MD simulation for the bound CS06.

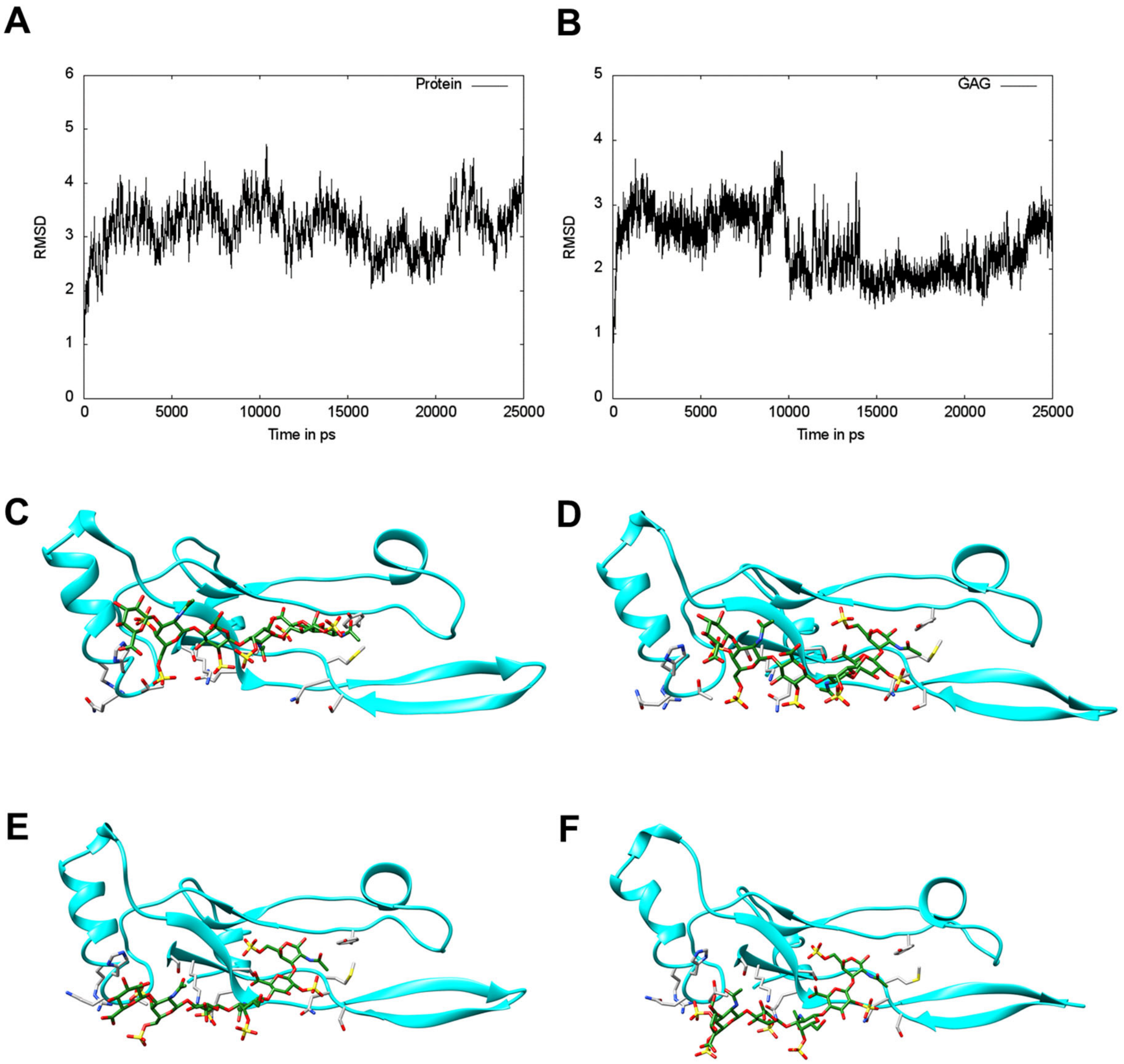

Figure S4. Conformational sampling of CS06 bound in BS2 of TGF-β2 from MD simulation to give an overall picture on the movement of protein (A) and ligand (B) was in presence of solvent (see **Movie SM2**). (C) through (F) show representative frames of the MD simulation showing the bound hexasaccharide (in green sticks) and the interacting residues (shown as sticks). See Methods for details.

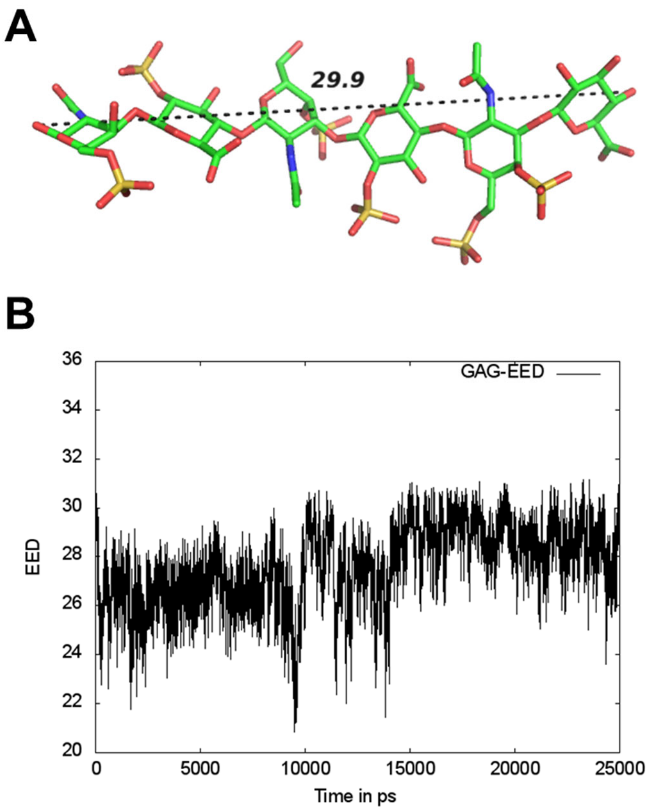

Figure S5. Conformational space exploration of TGF-β2-CS06 complex in BS2. A) A representative structure showing the end-to-end distance (EED) of the CS06. B) Time based fluctuation of the EED during the MD simulation for the bound CS06.

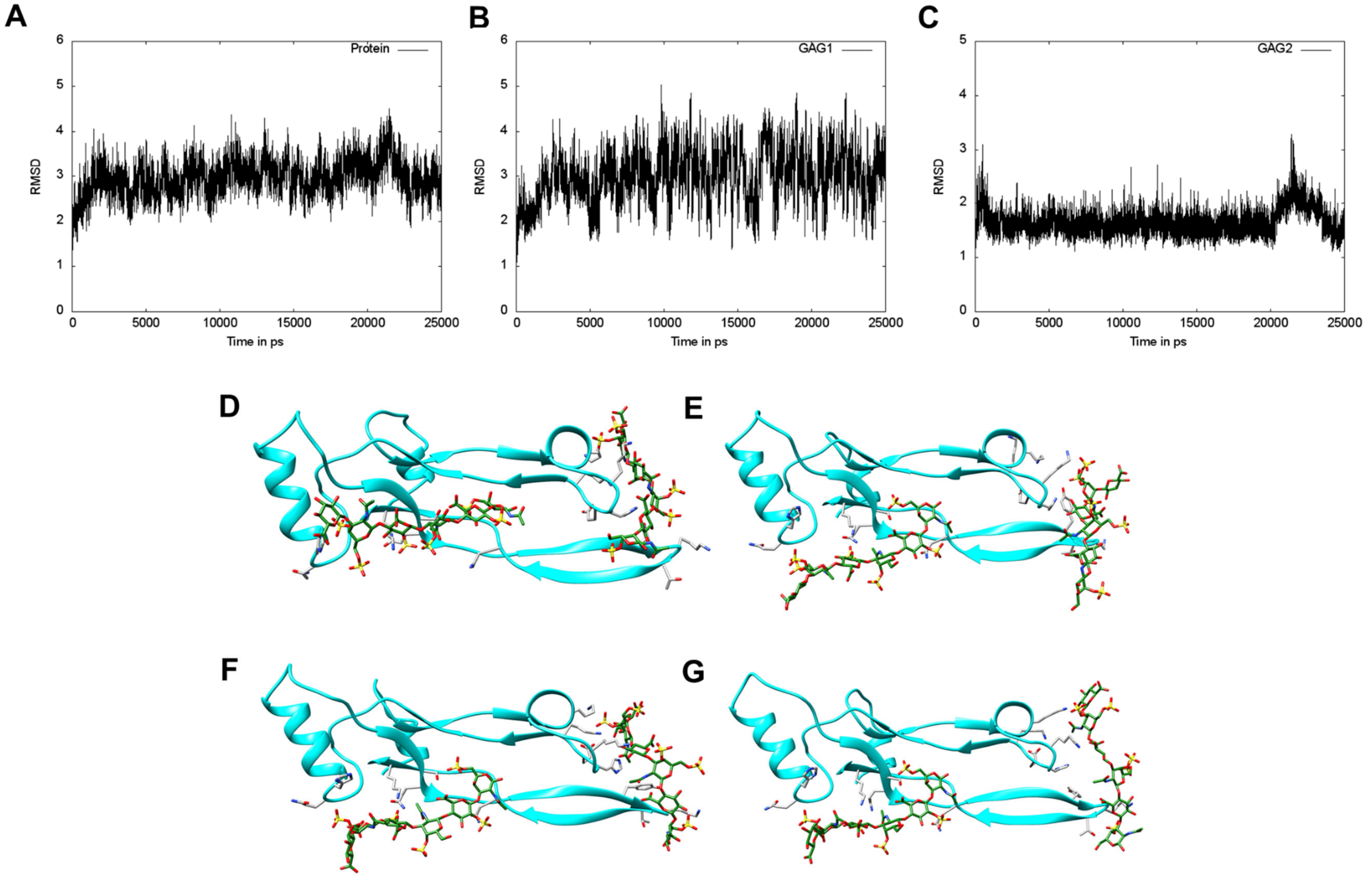

Figure S6. Conformational sampling of two CS06 sequences simultaneously bound in BS1 and BS2 of TGF-β2 from MD simulation to give an overall picture on the movement of protein (A) and ligand (B) was in presence of solvent (see **Movie SM3**). (C) through (F) show representative frames of the MD simulation showing the bound hexasaccharides (in green sticks) and the interacting residues (shown as sticks). See Methods for details.

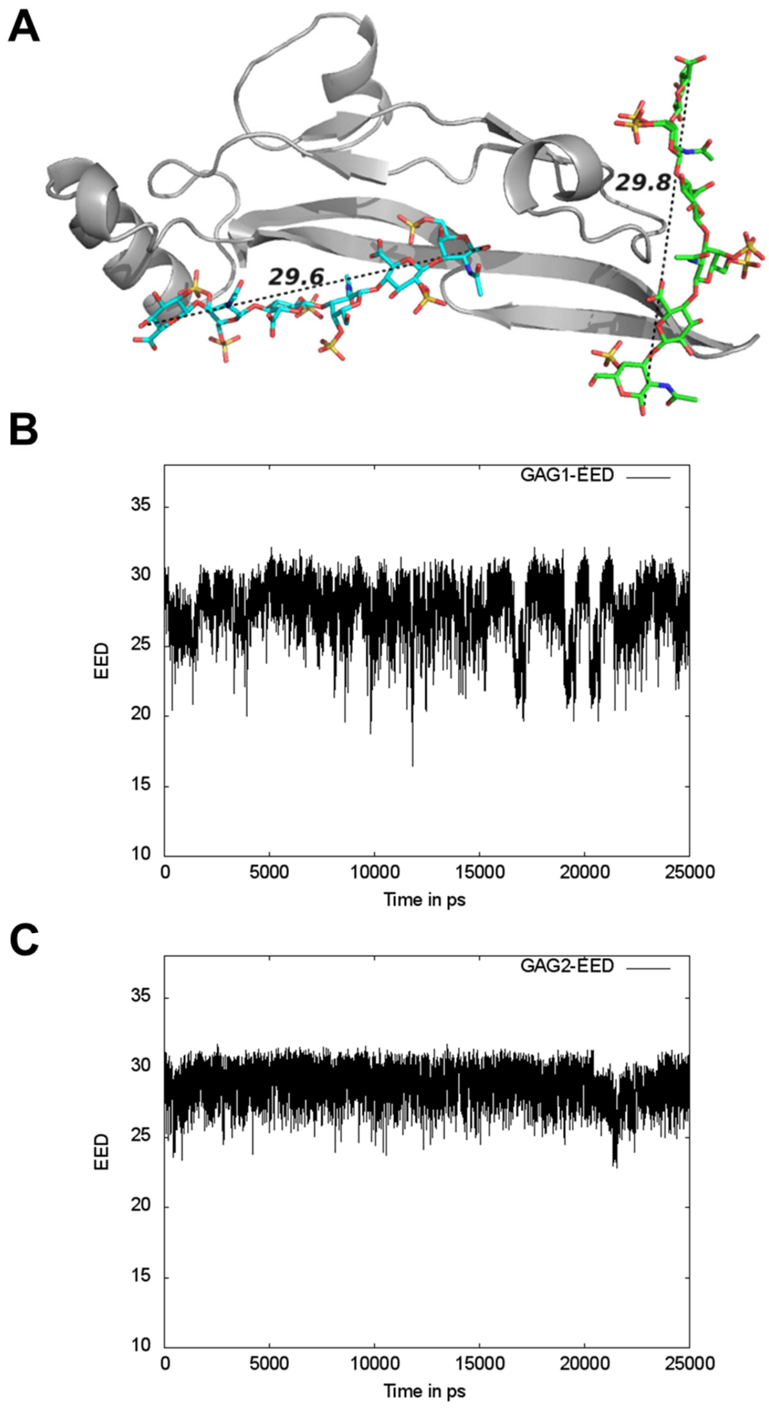

Figure S7. Conformational sampling of two CS06 sequences simultaneously bound in BS1 and BS2 of TGF-β2. A) A representative structure showing the end-to-end distance (EED) of the CS06. B) Time based fluctuation of the EED during the MD simulation for the bound CS06.
