# Supplementary figures and images for: Combinatorial Virtual Library Screening Study of Transforming Growth Factor-β2–Chondroitin Sulfate System
Source: Int J Mol Sci. 2021 Jul 14;22(14):7542. doi: 10.3390/ijms22147542 (PMC8305211; doi:10.3390/ijms22147542)

## Slide 1
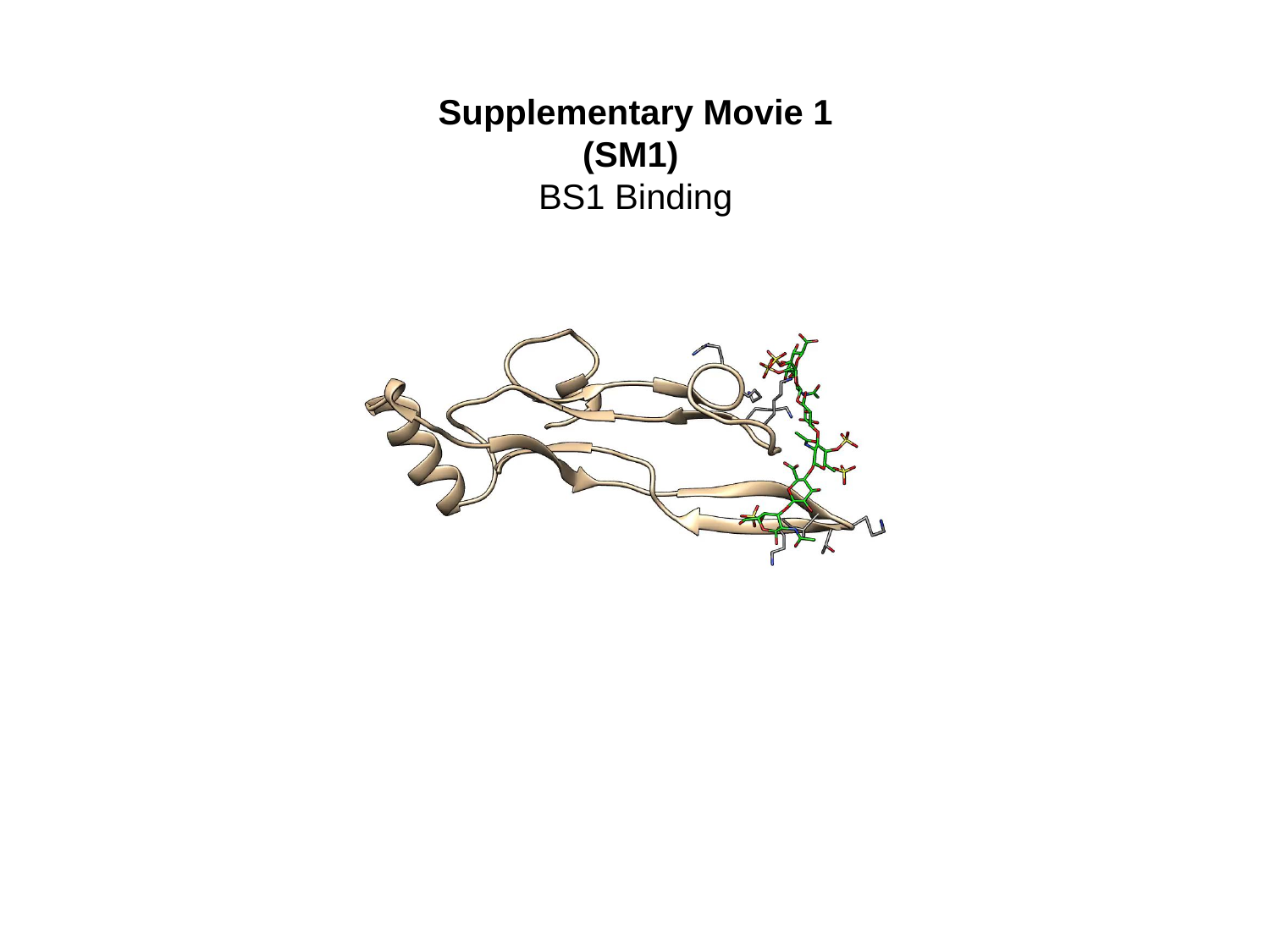

Supplementary Movie 1
(SM1)
BS1 Binding

Supplement: Supplementary file 1 [file ijms-22-07542-s001.zip › SupplMovie SM1.pptx]

## Slide 1
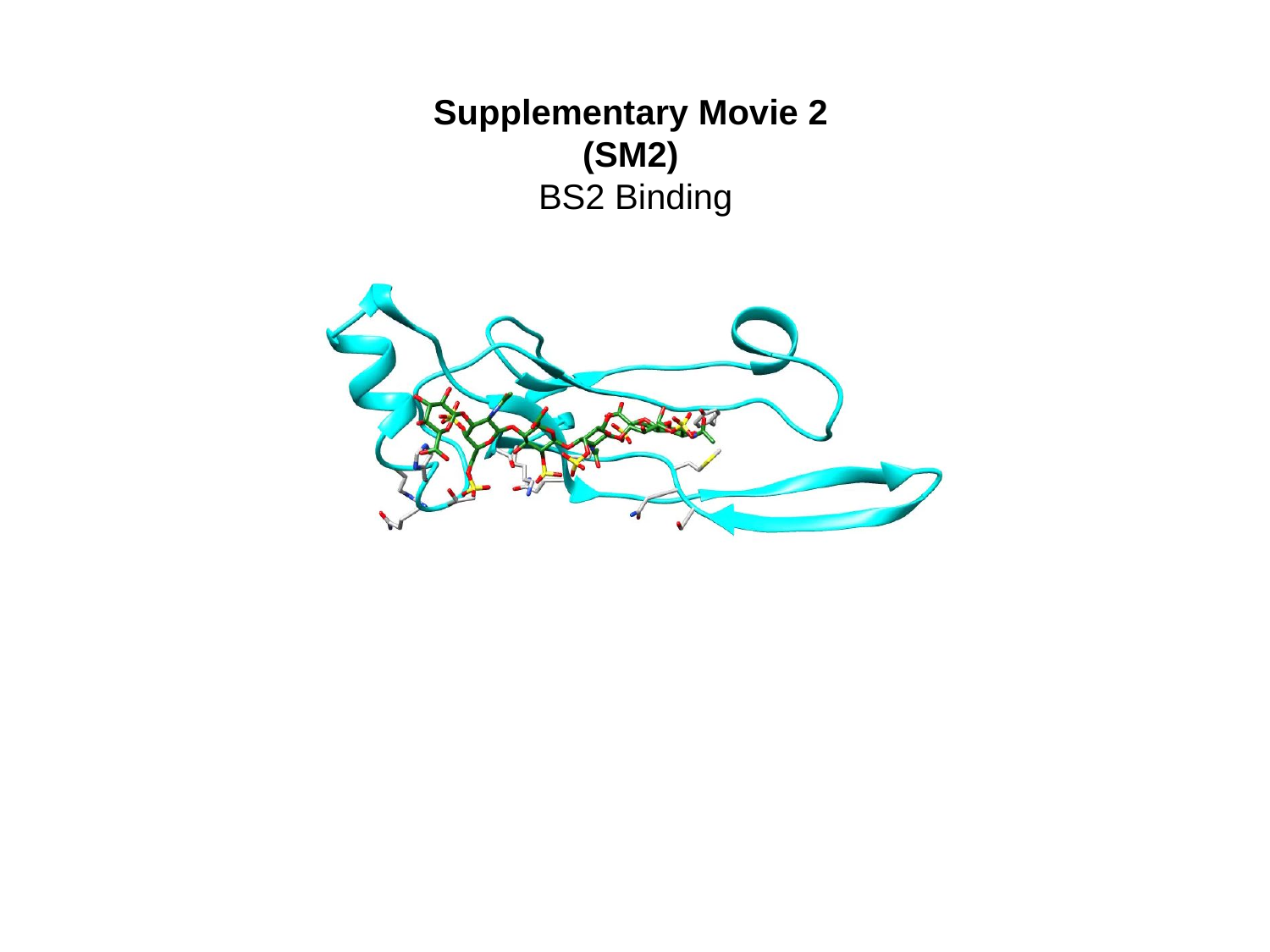

Supplementary Movie 2
(SM2)
BS2 Binding

Supplement: Supplementary file 1 [file ijms-22-07542-s001.zip › SupplMovie SM2.pptx]

## Slide 1
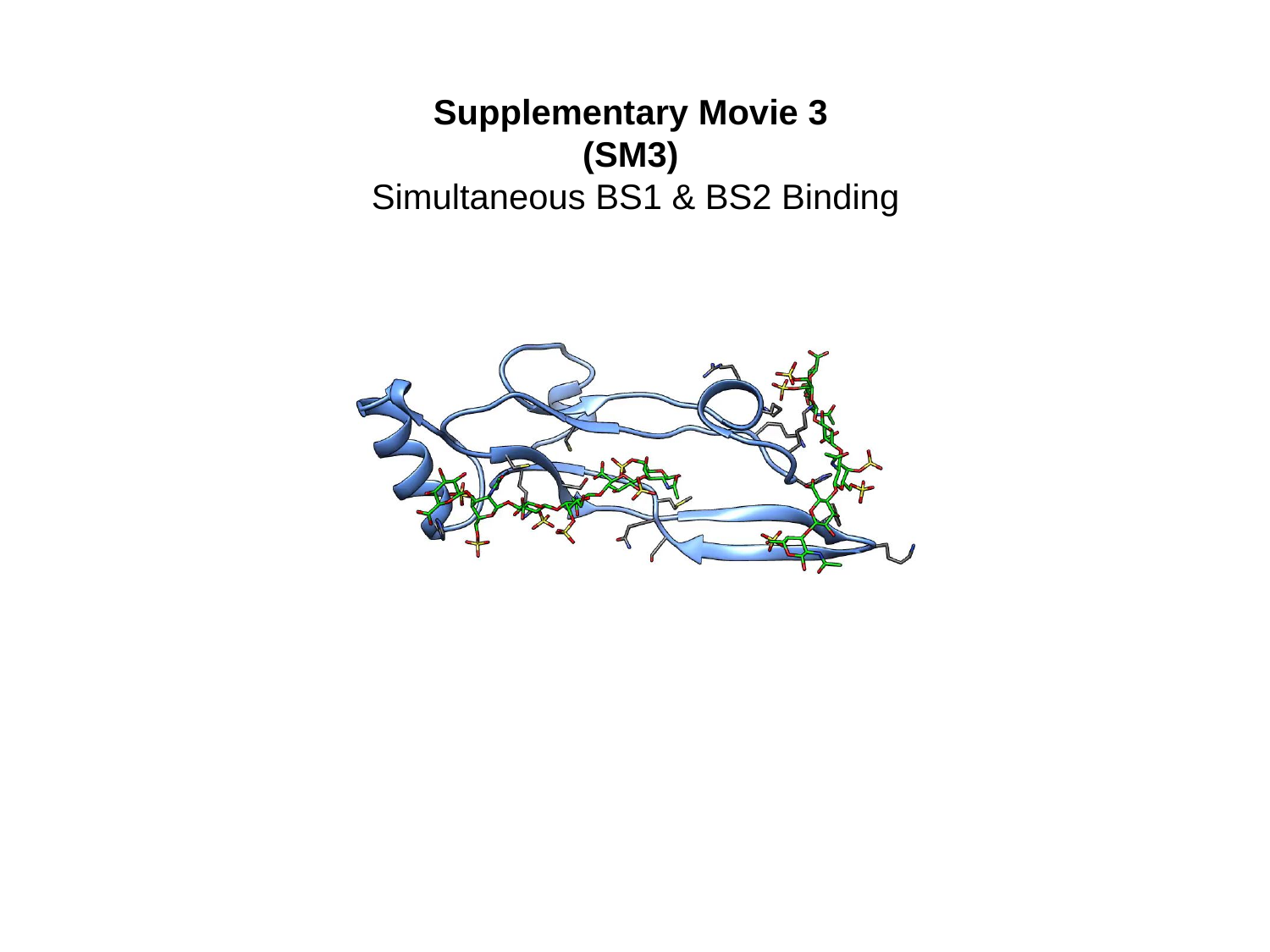

Supplementary Movie 3
(SM3)
Simultaneous BS1 & BS2 Binding

Supplement: Supplementary file 1 [file ijms-22-07542-s001.zip › SupplMovie SM3.pptx]
